# Editorial Note: Machine learning driven optimization of compressive strength of 3D printed bio polymer composite material

**DOI:** 10.1371/journal.pone.0339818

**Published:** 2026-01-06

**Authors:** The PLOS One Editors

The *PLOS One* Editors issue this Editorial Note to inform readers of concerns regarding compliance with PLOS Authorship policy for this article [1]. We regret that the issues were not addressed prior to the article’s publication.
